# TIC10/ONC201 synergizes with Bcl-2/Bcl-xL inhibition in glioblastoma by suppression of Mcl-1 and its binding partners *in vitro* and *in vivo*

**DOI:** 10.18632/oncotarget.5505

**Published:** 2015-10-12

**Authors:** Georg Karpel-Massler, Maïmouna Bâ, Chang Shu, Marc-Eric Halatsch, Mike-Andrew Westhoff, Jeffrey N. Bruce, Peter Canoll, Markus D. Siegelin

**Affiliations:** ^1^ Department of Pathology & Cell Biology, Columbia University Medical Center, New York, New York, U.S.A; ^2^ Department of Neurosurgery, Columbia University Medical Center, New York, New York, U.S.A; ^3^ Department of Neurosurgery, Ulm University Medical Center, Ulm, Germany; ^4^ Department of Pediatrics and Adolescent Medicine, Ulm University Medical Center, Ulm, Germany

**Keywords:** glioblastoma, apoptotic resistance, TIC10/ONC201, ABT263, multi-targeting

## Abstract

Glioblastoma is the most frequent primary brain tumor in adults. Current therapeutic options are sparse and the prognosis of patients suffering from this disease is grim. Abundance in intratumoral heterogeneity among different deregulated signaling pathways is a hallmark of glioblastoma and likely accounts for its recurrence and resistance to treatment. Glioblastomas harbor a plethora of deregulated pathways driving tumor formation and growth. In this study, we show that TIC10/ONC201, a promising compound that is currently in planned clinical development, along with Bcl-2/Bcl-xL inhibition by ABT263 yields a strong synergistic antiproliferative effect on pediatric, adult, proneural glioblastoma and glioma stem-like cells. On the molecular level, treatment with TIC10/ONC201 results in a posttranslational decrease of the anti-apoptotic Bcl-2 family member, myeloid cell leukemia 1 (Mcl-1), through modulation of the chaperone Bag3 and the deubiquitinase Usp9X. Consistently, the combination treatment of TIC10/ONC201 and ABT263 required the presence of functional BAX and BAK to drive intrinsic apoptosis, but is surprisingly independent of the extrinsic apoptotic pathway. Moreover, the expression of Noxa protein was required for efficient apoptosis induction by TIC10/ONC201 and ABT263. Importantly, the drug combination of TIC10/ONC201 and the BH3-mimetic, ABT263, led to a regression of tumors *in vivo*, without any notable toxicity and side effects. Overall, TIC10/ONC201 along with Bcl-2/Bcl-xL inhibition holds significant promise as a novel potential approach for the treatment of recalcitrant tumors such as glioblastoma.

## INTRODUCTION

For many solid tumor entities including glioblastoma, efficient therapeutic strategies still do not exist. Despite vast efforts, current standard of care provides glioblastoma patients only with a negligible survival benefit [1, 2]. With the advancement of modern molecular techniques, our understanding of cancer biology has rapidly grown and points out that intratumoral heterogeneity represents one driving force for the development of therapeutic resistance [3]. It seems unlikely that concomitant activation of different pro-neoplastic signaling pathways, which furthermore vary among different cancer cells within the same tumor, will be successfully addressed by one “magic bullet”. From that perspective, a multi-targeted therapeutic approach seems to hold more promise.

TRAIL-inducing compound 10 (TIC10/ONC201) has been identified by a small molecules screen, based on the National Cancer Institute chemical library, searching for compounds inducing tumor necrosis factor-related apoptosis-inducing ligand (TRAIL) in cancer cells [4]. In this study, Allen et al. showed that treatment with TIC10/ONC201 resulted in dual inhibition of AKT and ERK signaling followed by dephosphorylation of Foxo3a, subsequent nuclear translocation and enhanced transcription of TRAIL. In preclinical studies, TIC10/ONC201 was shown to inhibit various pro-neoplastic features of different cancer types such as breast cancer, colorectal cancer as well as lung cancer [4–7]. In addition, TIC10/ONC201 provides several favorable characteristics with respect to a potential future clinical application such as its capacity to cross the blood-brain barrier and a preserved activity when administered orally [8]. These features also render the compound particularly interesting for combinatorial therapeutic strategies targeting brain tumors.

Among the most significant regulators of programmed cell death are the members of the B-cell lymphoma-2 (Bcl-2) family of proteins [9]. Life or death of a cancer cell relies on the levels of each Bcl-2 family member [10]. Pro-apoptotic Bcl-2 family proteins, such as BAX, BAK, BAD, BIM, BID, NOXA and PUMA, facilitate death through several mechanisms, including direct activation of BAX or antagonization of anti-apoptotic Bcl-2 family members. Pro-apoptotic Bcl-2 family proteins share a conserved dimerization motif called Bcl-2 homology 3 (BH3) and stood model for small-molecule inhibitors such as ABT263, which were molecularly engineered to target anti-apoptotic Bcl-2 family proteins including Bcl-2, Bcl-xL, Bcl-w, but not Mcl-1. These so-called BH-3 mimetics have been successfully applied in early stage clinical trials for the treatment of lymphoid malignancies or small-cell lung cancer [11–14]. The major drawback of ABT-compounds is represented by the inability to bind and interfere with Mcl-1 function. Consequently, high levels of Mcl-1 are causal for resistance against ABT737, ABT263 and ABT199.

In this study, we tested the hypothesis whether TIC10/ONC201 sensitizes for inhibition of Bcl-2/Bcl-xL through BH3-mimetics. Our data show that TIC10/ONC201, when combined with BH3-mimetics, yields a synergistic anti-proliferative and pro-apoptotic effect in a BAX/BAK-dependent manner across different glioblastoma cells. Moreover, *in vivo*, combined treatment with TIC10/ONC201 and the BH-3 mimetic ABT263 results in a significant enhancement of tumor growth inhibition in a heterotopic glioblastoma model. From a mechanistic point of view, we identified down-regulation of Usp9X as well as Bag3 followed by enhanced Mcl-1-degradation as one of the driving mechanisms subjacent to the profound anti-cancer activity inherent to a combined inhibition of ERK signaling and Bcl-2/Bcl-xL.

## RESULTS

### TIC10/ONC201 along with Bcl-2/Bcl-xL inhibition yields a synergistic anti-proliferative effect

**Figure 1:**
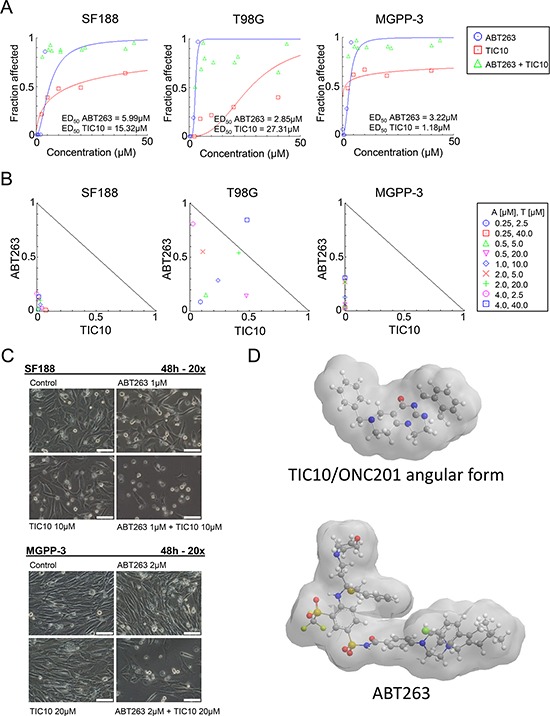
Combined treatment with the BH3-mimetic ABT263 and the ERK inhibitor TIC10/ONC201 yields a synergistic antiproliferative effect across different glioblastoma cells **A.** SF188 (pediatric), T98G (adult) and MGPP-3 (murine, transgenically derived) glioblastoma cells were treated with the indicated concentrations of TIC10/ONC201 or ABT263 under serum starvation (1.5% FBS). After 72 h, a MTT assay was performed. Graphs were plotted using the CompuSyn software. **B.** The antiproliferative effect of ABT263 and TIC10/ONC201 was assessed by an MTT assay after 72 h of treatment with the single agents or the respective combination at indicated concentrations under serum starvation (1.5% FBS). Normalized isobolograms were calculated using the CompuSyn software. The ED_50_ values of TIC10/ONC201 and ABT263 are normalized and plotted on the x- or y-axis. The connecting line represents additivity. Data points located below the line indicate a synergistic drug-drug interaction and data points above the line indicate an antagonistic drug-drug interaction. **C.** Representative microphotographs of SF188 and MGPP-3 glioblastoma cells treated with solvent, ABT263, TIC10/ONC201 or both for 48 h at indicated concentrations. Magnification, x20; scale bar, 100 μm. **D.** 3-dimensional representation of the chemical structures of TIC10/ONC201 and ABT263.

In order to examine whether TIC10/ONC201 along with inhibition of Bcl-2/Bcl-xL results in a mutually enhanced anti-proliferative effect, we performed MTT assays in pediatric (SF188), adult (T98G) and proneural (MGPP-3 - derived from a transgenic mouse model) glioblastoma cells. As shown in Figure 1A, we first determined the median effective dose (ED_50_) for the BH-3 mimetic ABT263 and the dual AKT/ERK inhibitor TIC10/ONC201 (Figure 1D) in all three cell lines. Next, we examined how the combined treatment with both compounds affects the proliferation of glioblastoma cells when compared to single-agent treatments. The dose-effect relationship for the combination of both agents was calculated based on the Chou-Talalay method generating normalized isobolograms (Figure 1B) and combination indices (Table 1). Our data show that treatment with ABT263 and TIC10/ONC201 results in a synergistic anti-proliferative effect (Figure 1B). These findings are illustrated by representative microscopic images of SF188 and MGPP-3 glioblastoma cells showing a marked reduction in cellular density for those cells subjected to the combination treatment (Figure 1C).

**Table 1:** Combined treatment with ABT263 and TIC10/ONC201 results in a synergistic anti-proliferative effect

SF188			T98G			MGPP-3		
ABT263 [μM]	TIC10 [μM]	CI	ABT263 [μM]	TIC10 [μM]	CI	ABT263 [μM]	TIC10 [μM]	CI
0.25	2.5	0.03301	0.25	2.5	0.17741	0.25	2.5	0.04539
0.25	40.0	0.08895	0.25	40.0	1.21740	0.25	40.0	0.02942
0.5	5.0	0.03954	0.5	5.0	0.28815	0.5	5.0	0.06144
0.5	20.0	0.07298	0.5	20.0	0.61698	0.5	20.0	0.06163
1.0	10.0	0.09260	1.0	10.0	0.52434	1.0	10.0	0.12883
2.0	5.0	0.13236	2.0	5.0	0.66104	2.0	5.0	0.26860
2.0	20.0	0.13319	2.0	20.0	0.95263	2.0	20.0	0.26916
4.0	2.5	0.16678	4.0	2.5	0.83819	4.0	2.5	0.30820
4.0	40.0	0.15353	4.0	40.0	1.32382	4.0	40.0	0.30820

The CompuSyn software (ComboSyn, Inc., Paramus, NJ, U.S.A.) was used for the drug-drug interaction analysis including the calculation of the combination index (CI). A CI < 1 was considered as synergistic, a CI = 1 as additive and a CI > 1 as antagonistic

### The combination treatment of TIC10/ONC201 and ABT263 enhances apoptosis and caspase-activation

**Figure 2:**
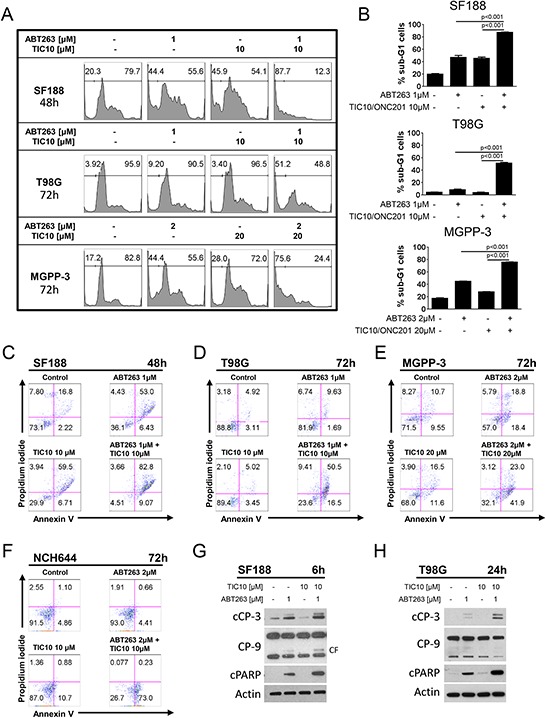
Combined treatment with ABT263 and TIC10/ONC201 results in an enhanced induction of apoptosis **A.** representative histograms of SF188, T98G and MGPP-3 glioblastoma cells that were treated as indicated with TIC10/ONC201, ABT263, both or solvent prior to staining with propidium iodide (PI) and flow cytometric analysis. **B.** quantitative representation of the fraction of sub-G1 cells for SF188, T98G and MGPP-3 cells treated as described for A. Columns, means of the fraction of sub-G1 cells. Bars, SD. **C–F.** representative histograms of SF188 (C), T98G (D), MGPP-3 (E) glioblastoma cells and NCH644 (F) glioma stem-like cells stained for annexin V/PI and treated as indicated. **G–H.** SF188 (G) and T98G (H) glioblastoma cells were treated for 6 h or 24 h respectively with TIC10/0NC201, ABT263 both agents or solvent under serum starvation (1.5% FBS). Whole-cell extracts were examined by Western blot for cleaved caspase 3 (cCP-3) or caspase 9 (CP-9 - CF = cleaved fragment) and cleaved PARP (cPARP). Actin Western blot analysis was performed to confirm equal protein loading.

We next assessed whether the synergistic anti-proliferative effect of ABT263 and TIC10/ONC201 is related to enhanced induction of apoptosis. Therefore, staining for propidium iodide was performed prior to flowcytometric analysis. As shown in Figure 2A and 2B, combined treatment with ABT263 and TIC10/ONC201 results in a statistically significant increase in the fraction of sub-G1 cells (apoptotic cells). In addition, we performed staining for annexin V/PI as an independent second and more apoptosis-specific method (Figure 2C–2F, Supplementary Figure 1). Combined treatment with ABT263 and TIC10/ONC201 results in a marked increase in the fraction of annexin V-positive (apoptotic) SF188 (pediatric - C), T98G (adult - D), MGPP-3 (transgenic proneural - E) and NCH644 (glioma stem-like - F) as well as NCH421K (glioma stem-like - Supplementary Figure 1) glioblastoma cells. In concordance with these findings, our data show enhanced cleavage of caspases 9, 3 and PARP following treatment with the combination of TIC10/ONC201 and ABT263 providing additional proof for enhanced induction of apoptosis due to this treatment on the molecular level (Figure 2G and 2H).

### Combined treatment with TIC10/ONC201 and ABT263 results in down-regulation of ERK signaling in glioblastoma

**Figure 3:**
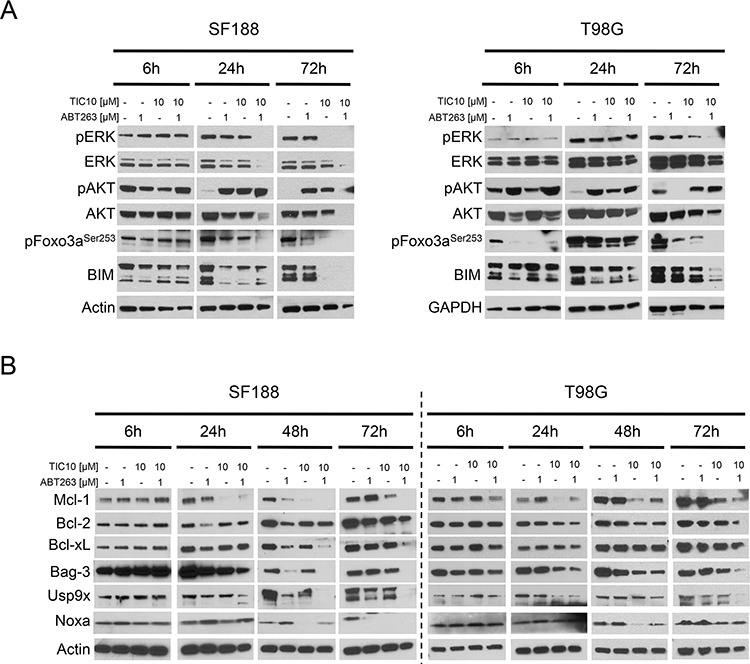
Combined treatment with ABT263 and TIC10/ONC201 yields down-regulation of ERK signaling as well as of the Mcl-1/Bag3/Usp9X network **A.** SF188 and T98G glioblastoma cells were treated for indicated lengths of time with ABT263, TIC10/ONC201, both agents or solvent under serum starvation. Whole-cell extracts were examined by Western blot for pERK, ERK, pAKT, AKT, pFoxo3a and BIM. Actin Western blot analysis was performed to confirm equal protein loading. **B.** SF188 and T98G glioblastoma cells were treated as described for A prior to collecting whole-cell extracts and performing Western blot analysis for Mcl-1, Bcl-2, Bcl-xL, Bag3, Usp9X and Noxa. Actin served as a loading control.

Previous data showed that treatment with TIC10/ONC201 resulted in dual inhibition of ERK and AKT [4]. To examine whether this finding holds also true for the setting of glioblastoma and furthermore, how it may be affected by an additional treatment with a BH3-mimetic, Western blot analyses were performed (Figure 3A). Our data show that combined treatment with both compounds results in an earlier and more pronounced dephosphorylation of ERK when compared to treatment with TIC10/ONC201 alone. Interestingly, pAKT levels were enhanced upon treatment with the combination which was in contrast to total AKT levels showing a decrease in expression after 24 h and 72 h of treatment with both compounds. Dephosphorylation of Foxo3a is a known downstream effect of dual inhibition of ERK and AKT. Despite a lack of a significant decrease in the expression of pAKT after treatment with TIC10/ONC201 alone or in combination with ABT263, expression of the phosphorylated form of Foxo3a was drastically reduced and more pronounced following a treatment with the combination. Protein expression levels of Bcl-2-like protein 11 (BIM), a pro-apoptotic Bcl-2 family member regulated by Foxo3a, showed an early increase and at later time points a marked decrease.

### Treatment with TIC10/ONC201 results in suppression of the anti-apoptotic Bcl-2 family member Mcl-1

Given the strong pro-apoptotic synergism of a combined treatment with TIC10/ONC201 and ABT263, we decided to further elucidate the subjacent molecular mechanism by putting the spotlight onto the mitochondrial pathway. Bcl-2 family proteins are important regulators of this pathway and its anti-apoptotic members are known mediators of apoptosis resistance. Expression of the anti-apoptotic Bcl-2 family member Mcl-1 represents one of the best described mechanisms of resistance towards ABT-compounds. As shown in Figure 3B and Supplementary Figure 2, treatment with ABT263 resulted in an early up-regulation of Mcl-1. In contrast, treatment with TIC10/ONC201 yielded a suppression of Mcl-1 which was maintained in the combination treatment. Western blot analyses of other anti-apoptotic Bcl-2 family members, Bcl-2 and Bcl-xL, showed inconsistent results. However, in both, SF188 and MGPP-3 glioblastoma cells Bcl-xL levels were depleted when cells were treated with the combination treatment. Moreover, in MGPP-3, combined treatment with both compounds resulted in a significant reduction in Bcl-2 expression.

### Treatment with TIC10/ONC201 and concomitant inhibition of Bcl-2/Bcl-xL depletes the Mcl-1 chaperone Bag3 and the deubiquitinase Usp9X

To further dissect by which means Mcl-1 expression is mechanistically affected we performed Western blot analyses for Bag3 and Usp9X - two molecules that are known to affect the stability of Mcl-1. Both, Bag3 and Usp9X expression levels were significantly decreased when cells received treatment with TIC10/ONC201 alone suggesting that the inhibitory effect of TIC10/ONC201 on Mcl-1 is at least partly mediated through an inhibition of Bag3 and Usp9X (Figure 3B, Supplementary Figure 2). Moreover, combined treatment with TIC10/ONC201 and ABT263 lead to an even stronger down-regulation of Bag3 and Usp9X.

### Down-regulation of Mcl-1 is not transcriptionally mediated but due to enhanced proteasomal degradation

**Figure 4:**
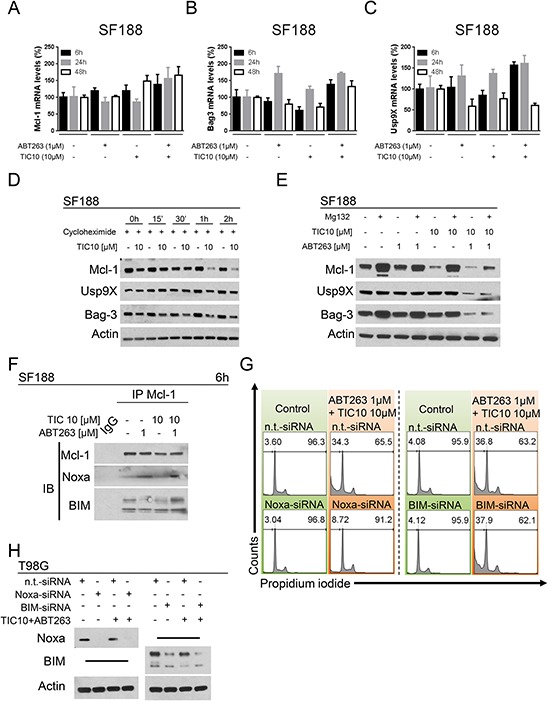
Down-regulation of Mcl-1 is mediated through enhanced proteasomal degradation **A.** SF188 glioblastoma cells were treated for 6 h, 24 h or 48 h with ABT263, TIC10/ONC201, both agents or solvent under serum starvation prior to collecting RNA and performing rtPCR for MCl-1 (A), Bag3 (B) or Usp9X (C) Columns, means of the percentage of mRNA expression normalized to control. Bars, SD. **D.** SF188 glioblastoma cells were treated with the protein synthesis inhibitor cycloheximide (10 μg/ml) in the presence or absence of TIC10/ONC201 for the indicated lengths of time. Western blot analysis was performed for Mcl-1, Usp9X and Bag3. Actin expression was determined to confirm equal protein loading. **E.** SF188 pediatric glioblastoma cells were treated with ABT263, TIC10/ONC201, both or solvent in the presence or absence of the proteasome inhibitor MG132 (10 μM) for 5 h. Whole-cell extracts were collected prior to Western blot analysis for Mcl-1, Usp9X and Bag3. Actin expression was determined to confirm equal protein loading. **F.** SF188 glioblastoma cells were treated for 6 h with ABT263, TIC10/ONC201, both agents or solvent under serum starvation. Whole-cell extracts were collected prior to immunoprecipitation (IP) for Mcl-1. IP with murine non-specific IgG served as negative control. Western blot analysis for Mcl-1, Noxa and BIM was performed for the immunoprecipitate. **G.** T98G glioblastoma cells were treated with non-targeting (n.t.)-siRNA, Noxa-siRNA or BIM-siRNA followed by a treatment with TIC10/ONC201/ABT263 or solvent for 24 h. Staining for propidium iodide was performed prior to flow cytometric analysis. Representative histograms are shown. **H.** T98G glioblastoma cells were treated as described for G. Whole-cell extracts were collected and analysed by Western blot for Noxa and BIM to confirm successful knock-down. Actin Western blot analysis was performed to ensure equal protein loading.

We next assessed whether the decreased protein expression of Mcl-1, Bag3 or Usp9X are a consequence of a transcriptional down-regulation. As shown in Figure 4A and 4B, Mcl-1 mRNA levels were not significantly reduced by the single-agent treatments and mRNA levels for Bag3 were only slightly reduced upon treatment with TIC10/ONC201 alone for 6 h and 48 h. However, when treated with the combination of both agents Mcl-1 and Bag3 mRNA levels were increased across all three time points. For Usp9X, mRNA levels were not decreased at early time points following single-agent treatments but at 48 h they were reduced to approximately 50–60% of control (Figure 4C). Combined treatment with both compounds resulted in an increase in mRNA levels for Usp9X except after 48 h where a 50% decrease was noted which however, did not exceed the decrease in Usp9X mRNA levels observed after treatment with ABT263 alone. Moreover, when blocking protein synthesis by treatment with cycloheximide, a marked reduction in protein stability was found for Mcl-1 when cells were treated with TIC10/ONC201 (Figure 4D). In addition, inhibition of proteasomal degradation by treatment with MG132 resulted in a significant restoration of Mcl-1 protein levels for cells treated with TIC10/ONC201 alone or in combination with ABT263 (Figure 4E).

### Noxa but not BIM is required for the efficient cell death induction by the combination treatment of TIC10/ONC201 and ABT263

Given that Mcl-1 was significantly down-regulated by TIC10/ONC201 and the combination of ABT263 and TIC10/ONC201, we next examined whether the pro-apoptotic Bcl-2 family members Noxa and BIM, which interact with Mcl-1, are required for cell death induction by the combination treatment. Therefore, we conducted immunoprecipitation studies to assess if the interaction of Noxa and BIM with Mcl-1 is regulated by the various treatments. Treatment with ABT263 alone and the combination of TIC10/ONC201 and ABT263 enhanced the association of Noxa with Mcl-1 (Figure 4F). The BIM – Mcl-1 interaction was mildly enhanced by the combination treatment. These results suggest that Noxa and BIM are potentially implicated in TIC10/ONC201/ABT263-mediated cell death. To further test this hypothesis we performed knock-down experiments for both BIM and Noxa (Figure 4G and 4H). While BIM silencing did not rescue the effect of the combination treatment, Noxa knock-down almost completely abrogated the pro-apoptotic effect of the combination treatment.

### Silencing of Mcl-1, Bag3 or Usp9X are sufficient to sensitize GBM cells to ABT263-mediated apoptosis

**Figure 5:**
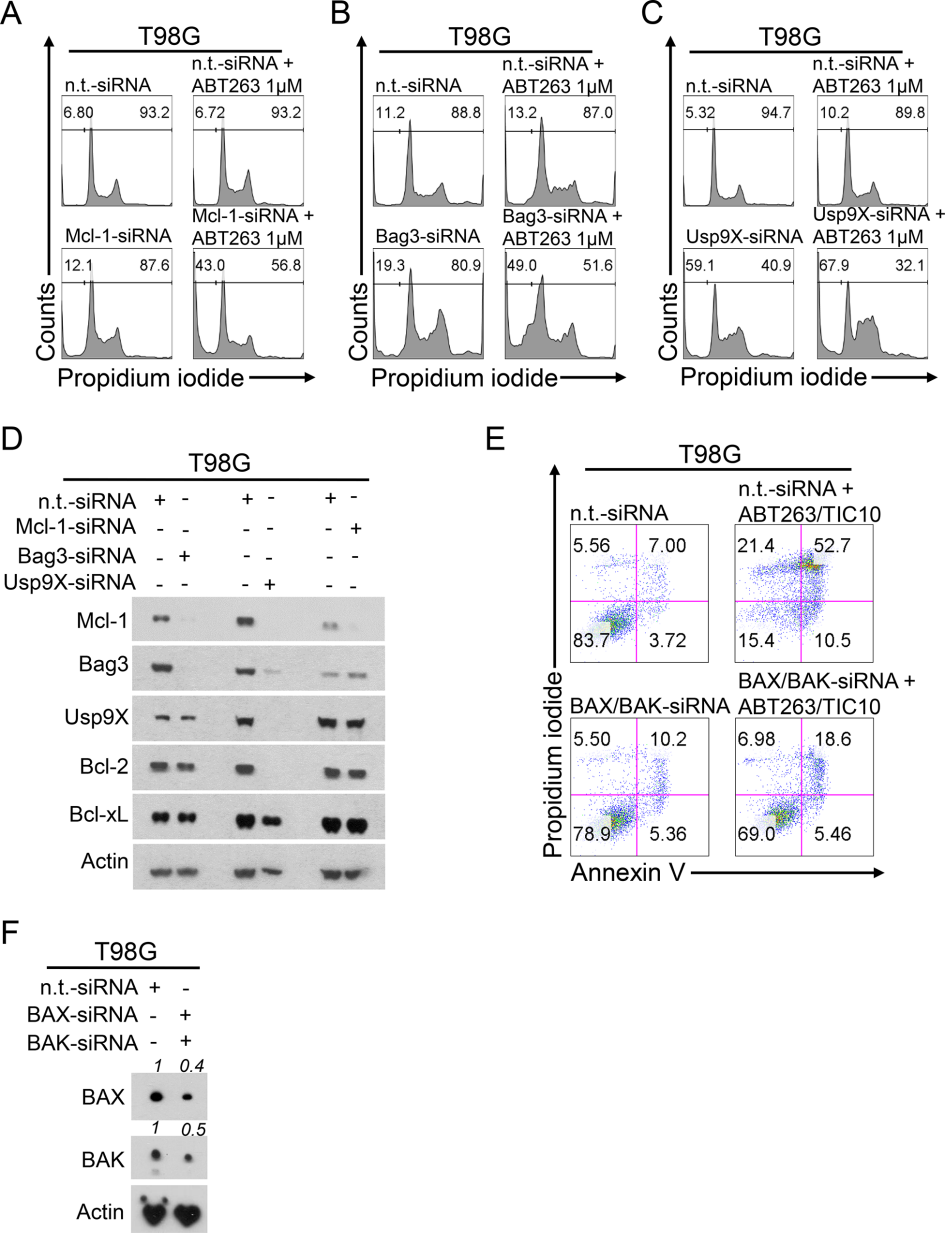
Down-regulation of Mcl-1, Bag3 or Usp9X sensitizes for ABT263-mediated apoptosis and double knock-down of BAX and BAK rescues from TIC10/ONC201/ABT263-mediated apoptosis **A–C.** representative histograms of T98G glioblastoma cells treated with non-targeting (n.t.)-siRNA or Mcl-1-siRNA (A), Bag3-siRNA (B) or Usp9X-siRNA (C) prior to treatment with ABT263 for 24 h. Then cells were stained with propidium iodide and subjected to flow cytometric analysis. The fraction of sub-G1 cells was determined. **D.** T98G glioblastoma cells were treated as described for A-C. Whole-cell extracts were collected and analysed by Western blot for Mcl-1, Bag3, Usp9X, Bcl-2 and Bcl-xL to confirm successful knock-down. Actin Western blot analysis was performed to ensure equal protein loading. **E.** representative histograms of T98G glioblastoma cells treated with n.t.-siRNA or BAX/BAK-siRNA prior to combined treatment with 1 μM ABT263 and 10 μM TIC10/ONC201 for 24 h and staining with annexin V/PI and flow cytometric analysis. **F.** T98G glioblastoma cells were treated as described for E. Whole-cell extracts were collected and analysed by Western blot for BAX and BAK to confirm successful knock-down. Densitometric analysis was peformed using the ImageJ software (National Institutes of Health, U.S.A., http://imagej.nih.gov/ij). Relative pixel density is presented. Actin Western blot analysis was performed to ensure equal protein loading.

In order to examine whether the biological effects of the combination treatment can be mimicked by a different means we performed siRNA experiments based on the observations we made on the molecular level. As shown in Figure 5A, knock-down of Mcl-1 alone resulted in a slight increase in the fraction of sub-G1 cells. However, when cells were treated in addition with ABT263 a significant additional increase in the fraction of apoptotic cells was noted. Similarly, knock-down of the Mcl-1 chaperone Bag3 or the deubiquitinase Usp9X, both known to stabilize Mcl-1, lead to a marked increase in the fraction of sub-G1 cells when combined with ABT263 (Figure 5B and 5C). Consistently, siRNA-mediated knock-down of either Usp9X or Bag3 resulted in a concomitant suppression of Mcl-1 protein levels (Figure 5D). These findings are also in concordance with the notion that the protein stability of Mcl-1 is reduced upon TIC10/ONC201 treatment (see Figure 4).

### The pro-apoptotic synergism of a combined treatment with TIC10/ONC201 and ABT263 depends on the presence of the multi-domain effector proteins BAX and BAK

Oligomerization of the multi-domain effector proteins BAX and BAK, subsequent pore formation and mitochondrial outer membrane permeabilization are hallmarks of apoptosis induction through the intrinsic pathway. To assess whether the presence of BAX and BAK are necessary for the synergistic pro-apoptotic effect exerted by a combined treatment with TIC10/ONC201 and ABT263, we performed knock-down experiments silencing both BAX and BAK simultaneously (Figure 5E and 5F). As shown in Figure 5E, combined knock-down of BAX and BAK yielded a nearly complete prevention of apoptosis which stresses the importance of the intrinsic pathway as the mechanistic route responsible for the pro-apoptotic synergism of the combination treatment.

### The pro-apoptotic synergism of TIC10/ONC201 and ABT263 is independent of extrinsic pathway activation

It has been previously shown that treatment with TIC10/ONC201 yields an enhanced transcriptional activity for TRAIL in cancer cells which in turn has been postulated to be responsible for part of the anti-neoplastic activity attributed to this compound [4]. We therefore examined whether activation of the extrinsic pathway contributes to the pro-apoptotic synergism of the combination treatment. T98G glioblastoma cells treated with siRNA against DR5, the main death receptor for TRAIL in glioblastoma, showed no significant attenuation of apoptosis induced by a combined treatment with ABT263 and TIC10/ONC201 when compared to cells not silenced for DR5 (Supplementary Figure 3A and 3B). Moreover, treatment with caspase 8-siRNA (Supplementary Figure 3A and 3C) or the caspase 8 inhibitor Z-IETD-FMK (Supplementary Figure 3D) did not provide a protection against the pro-apoptotic effect of the combination treatment.

### Treatment with TIC10/ONC201 enhances the anti-cancer activity of ABT263 *in vivo*

**Figure 6:**
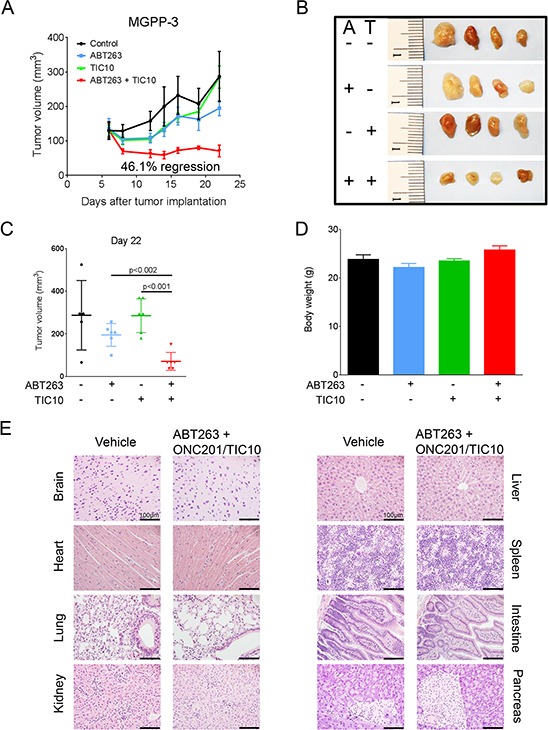
Combined treatment with ABT263 and TIC10/ONC201 results in an enhanced inhibition of tumor growth *in vivo* 5 × 10^5^ MGPP-3 glioblastoma cells were implanted subcutaneously. After tumor formation animals were treated intraperitoneally with vehicle (*n* = 5 tumors), ABT263 (25 mg/kg; *n* = 6 tumors), TIC10/ONC201 (25 mg/kg; *n* = 6 tumors) or both agents (*n* = 6 tumors) 3 times a week for 2 weeks. **A.** tumor growth curves showing the increase in tumor size for each treatment group. Data are presented as mean and SEM. **B.** representative photographs of the tumors; A = ABT263, T = TIC10/ONC201. **C.** quantification and statistical analysis (Student's *t*-test) of the tumors of the different treatment groups 22 days after tumor implantation. **D.** graphical representation of the bodyweight of the animals on day 22. Data are presented as mean and SEM. **E.** representative microphotographs showing the histological morphology (H & E staining) of the indicated organs among representative animals receiving treatment either with vehicle or the combination of ABT263 and TIC10/ONC201. Magnification, x40; scale bar, 100 μm.

To assess the therapeutic efficacy of this combination therapy *in vivo*, 5 × 10^5^ PDGF+, PTEN−/−, p53−/−, luciferase+ (MGPP-3) glioblastoma cells that were derived from a proneural transgenic mouse model were implanted subcutaneously as a 1:1 suspension in matrigel. Once tumors formed, the mice were randomized and treated with ABT263 (25 mg/kg), TIC10/ONC201 (25 mg/kg), both agents or solvent. Under these conditions, animals that received the combination treatment had statistically significantly smaller tumors than animals treated with vehicle, ABT263 or TIC10/ONC201 alone (Figure 6A–6C). Remarkably, animals treated with the drug combination showed a 46.1% tumor regression, indicating that the combination therapy did not only affect tumor proliferation but also impacted tumor cell death *in vivo* (Figure 6A). This notion is reinforced by the fact that histological analysis showed enhanced TUNEL staining in tumors derived from animals subjected to the combination therapy (Supplementary Figure 4). We did not detect any significant weight loss across the different treatment groups (Figure 6D). Consistently, histological analysis showed no tissue alterations in brain, lung, kidney, heart, liver, spleen, intestine or pancreas that may indicate organo-toxic effects (Figure 6E).

## DISCUSSION

Glioblastoma remains to be a major therapeutic challenge. Intra-tumoral heterogeneity is characterized by an activation of several oncogenic tumor driver pathways in different cells within the same tumor and represents one of the major obstacles for a therapeutic success in glioblastoma. While certain malignancies, such as chronic myeloid leukemia, have shown response to a single-agent treatment, this seems unlikely in the setting of glioblastoma given the implications described above. Therefore, alternative strategies are necessary. One key strategy would be to target multiple deregulated pathways by drug combination therapies. The effects of each compound may be remarkably enhanced when combining even just two drugs - ideally in a synergistic manner and without increasing side effects.

Here, we identified a novel drug combination that has not been described in any tumor entity thus far. Alike other tumor entities, glioblastoma displays a remarkable resistance against programmed cell death. There are different reasons why tumors acquire resistance towards apoptosis. The activation of multiple oncogenic pathways certainly contributes to this phenomenon. Consequently, drug combinations that simultaneously target these different pathways may represent a welcome contribution to the treatment battery for malignant brain tumors.

Our initial hypothesis was that dual targeting of the extrinsic and intrinsic apoptosis pathways might be a powerful approach to combat these tumors. In the past, enthusiasm about this strategy was largely dampened by the fact that inducers of extrinsic apoptosis bear highly unfavorable pharmacokinetics (recombinant human TRAIL) or in some cases even severe side effects, such as hepatotoxicity (FasL) [15]. Still, death ligands, such as TRAIL, demonstrated significant anti-glioma activity in several preclinical models of malignant glioma [16–18]. Moreover, it is notable that TRAIL-mediated apoptosis can be enhanced by various means, suggesting that TRAIL-based therapies are particularly effective in combination therapies. In most instances, combination therapies are studied in the setting of two drug compounds, but it may be worthwhile in the future to even combine more than two treatment modalities in order to elicit the most pronounced inhibition on tumor growth [19].

TIC10/ONC201 was introduced as a unique compound that induces TRAIL at the level of transcription through a Foxo3a-dependent mechanism [4]. This finding is considered a “breakthrough” since before its discovery extrinsic apoptosis induction was entirely centered on death-receptor agonistic antibodies, such as lexatumumab, or on genetically engineered recombinant human TRAIL (AA: 114–281). For our study, we decided to use the angular form of TIC10 since this form was shown to have TRAIL-inducing capacity [20]. In order to target the intrinsic apoptotic pathway we utilized a novel orally available BH3-mimetic, Navitoclax also designated as ABT263. Tagscherer et al. have initially proposed that inhibition of Bcl-2/Bcl-xL through ABT-compounds is a worthwhile strategy for the treatment of glial tumors [21]. More recently, the group led by Dent and colleagues has shown that the BH3-mimetic GX15-070 (Obatoclax) may be active in drug combination therapies in an orthotopic model of glioblastoma [22] and suggested that GX15-070 may be capable of crossing the blood-brain-barrier. In line with our initial hypothesis the drug combination of TIC10/ONC201 and ABT263 elicited strong synergistic cell death induction in adult and pediatric established GBM cells as well as in stem cell-like glioma cells.

Mechanistically, TIC10/ONC201 leads to a significant reduction in Mcl-1 protein levels along with its interacting proteins, Usp9X [23] and Bag3 [24]. Given the impact of Usp9X on the expression of anti-apoptotic Bcl-2 family members and the reduction of Usp9X by TIC10/ON201 or the combination treatment it is anticipated that the decline of Usp9X further facilitates the cell death induction mediated by the combination therapy. TIC10/ONC201-mediated reduction in Mcl-1 levels occurred in a posttranslational manner as protein stability of Mcl-1 was reduced upon TIC10/ONC201 treatment. This posttranslational reduction of Mcl-1 by TIC10/ONC201 may be related to its inhibitory effect on ERK since ERK was shown to phosphorylate Mcl-1 at Threonine 163 and thereby to regulate its turnover [25]. In the present literature numerous groups, including ours, have identified Mcl-1 as a key factor of resistance to BH3-mimetics [26–32]. This is explained by the notion that ABT-compounds mostly interfere with the function of Bcl-2 and Bcl-xL, while they have essentially no effect on Mcl-1 [33, 34]. Moreover, ABT263 and ABT737 have been reported to even increase the levels of Mcl-1 thereby driving paradoxical resistance. Therefore, strategies to interfere with Mcl-1 are currently explored such as the design of novel compounds that bind to Mcl-1 [30] or the discovery of molecules that suppress Mcl-1 levels in cancer cells [35]. While drug-mediated suppression of Mcl-1 levels in cancer cells takes place at multiple instances, commonly this involves reduction of protein stability of Mcl-1. However, other molecules have been shown to suppress Mcl-1 at the level of transcription as well as translation, such as mTORC1/2 inhibitors through modulation of 4EBP1. Moreover, TIC10/ONC201 caused a down-regulation of the Hsp70 co-chaperone Bag3, which has been reported to mediate resistance to BH3-mimetics in glioblastoma by regulating Mcl-1 levels. Consistently, siRNA-mediated knock-down of Bag3 significantly reduced Mcl-1 levels and sensitized T98G glioblastoma cells to ABT263. Earlier Bag3 was suggested as a therapeutic target for GBM [36].

Yet, knock-down of Bag3 only marginally induces cell death and from our own observation it appears that Mcl-1-dependent cell lines are particularly susceptible to Bag3 withdrawal. Regardless of Mcl-1-dependency specific knock-down of Bag3 sensitizes cancer cells to the cytotoxic effects of ABT263 and ABT737, suggesting that interference with Bag3 may be useful to chemo-sensitization.

In addition, we found that the combination treatment of TIC10/ONC201 and ABT263 regulated the expression levels of the deubiquitinase Usp9X. Previous data from others and our own group indicated that Usp9X plays a role in the regulation of Mcl-1 and consequently determines susceptibility of cancer cells to the sensitivity of BH3-mimetics [23, 28, 37–39].

Our data confirms the previous notion that knocking down Usp9X suppresses Mcl-1 along with Bcl-2 and primes glioblastoma cells to ABT263. Usp9X remains an interesting target as its levels are increased in glioblastoma and this molecule maintains high levels of certain anti-apoptotic factors, such as Mcl-1, Inhibitor of Apoptosis Proteins and a gene fusion observed in 40% of prostatic carcinomas [40].

With respect to pro-apoptotic Bcl-2 family members, it is known that BIM is sequestered by Mcl-1, leading to interference with its cell death inducing properties [41]. Therefore, down-regulation of Mcl-1 leads to liberation of BIM that in turn is either sequestered by Bcl-xL or engages directly in intrinsic apoptosis through binding and activation of the pro-apoptotic effector BAX [42, 43]. Moreover, inhibition of MEK has been shown to enhance BIM expression [41]. However, in our model system we appreciated only an early settle increase in BIM levels. Given that TIC10/ONC201 had a major impact on Mcl-1 levels this will likely result in an increase of unbound BIM. Another pro-apoptotic molecule is Noxa which is encoded by the *PMAIP1* gene. Noxa has been shown to negatively regulate Mcl-1 and when up-regulated to promote sensitivity to ABT compounds [44–47]. Factors that lead to an increase of Noxa often activate p53 or induce a stress response. TIC10/ONC201 did not regulate the expression of Noxa, but we saw a modulation of Noxa through ABT263. Therefore, we assessed whether specific suppression of Noxa may interfere with ABT263/TIC10/ONC201-mediated cell death. Our data show that Noxa is a major effector of this drug combination. We also tested whether the binding of BIM and Noxa to Mcl-1 is influenced by TIC10/ONC201, ABT263 or the combination of both. Under our conditions, we did not find an enhanced release of BIM from Mcl-1. Although there was no increased binding of Noxa to Mcl-1 in cells treated with TIC10/ONC201 alone, the combination treatment led to an increased association of Noxa and Mcl-1. Notably, the fact that BIM is not primarily involved in cell death mediated by ABT263 and TIC10/ONC201 may be also cell line-dependent. In addition, the requirement for BIM to induce cell death may be also related to the particular drug combination tested as certain reagents may depend more on the presence of BIM than others.

Since TIC10/ONC201 was reported to induce TRAIL and mediate extrinsic apoptosis we assessed [4–7] whether interference with caspase 8 and DR5 by specific siRNAs would attenuate death induced by the combination therapy of ABT263 and TIC10/ONC201. To our surprise, the combination therapy was neither dependent on caspase 8 nor on DR5. Moreover, the combination treatment was also active in NCH421K cells, which express very low levels of caspase 8 [48]. These results indicate that the combination of TIC10/ONC201 and ABT263 may have anti-glioma activity against tumors with absent or low levels of caspase 8. However, the observation that the combination treatment of TIC10/ONC201 and ABT263 does not depend on the presence of caspase 8 may be attributed to the fact that relatively low concentrations of TIC10/ONC201 were used which may not suffice to mediate a strong TRAIL induction. Another explanation may be that these observations are cell line-dependent and certain cell types may require less TIC10/ONC201 to elicit an extrinsic apoptotic response. Thus, Mcl-1 which sequesters a significant pool of BAK in cancer cells seems to be the major determinant for the sensitization of TIC10/ONC201 to ABT263-mediated apoptosis. In line with that assumption is our finding that the drug combination of TIC10/ONC201 and ABT263 induces apoptosis in a BAX/BAK-dependent manner since knock-down of BAX/BAK significantly abrogated cell death by the combination therapy in T98G glioblastoma cells.

An earlier study suggested that the drug combination of TRAIL and ABT737 might be efficient for the treatment of glioblastoma [49]. From a mechanistic point of view, combined treatment with TRAIL and ABT737 elicited BAX- as well as BID-dependent apoptosis since TRAIL was shown to activate caspase 8, leading to cleavage of BID to tBID and thereby to the activation and enhancement of intrinsic apoptosis induced by ABT737. Since in our setting, the effects of the combination treatment did not depend on caspase 8, we did not consider tBID as a major factor in TIC10/ONC201/ABT263-mediated apoptosis. Overall, these results reinforce the notion that down-regulation of Mcl-1 is the driving force for the sensitization of TIC10/ONC201 to ABT-compounds. However, we cannot exclude that under other conditions or in other model systems the extrinsic apoptotic pathway may contribute to the facilitation of ABT263-mediated apoptosis in the presence of TIC10/ONC201.

Finally, we examined whether the drug combination of TIC10/ONC201 and ABT263 is also effective *in vivo*. We therefore utilized a heterotopic proneural transgenic glioblastoma model. In this model, the drug combination led to a tumor regression, while the single and vehicle treatments did not cause a significant growth reduction. Notably, the drug combination did not cause any organ toxicity, suggesting that the treatment is feasible and may have little to no side effects. At this point, it has not entirely unraveled whether gliomas of proneural subtype are particularly prone or resistant to Bcl-2/Bcl-xL inhibition. This is currently being under investigation. Nevertheless, proneural gliomas encompass a significant amount of gliomas which also include lower-grade gliomas and secondary glioblastomas. Overall, these observations confirm that drug combination therapies, involving Bcl-2/Bcl-xL inhibition, are potentially feasible for the treatment of glioblastoma.

## MATERIALS AND METHODS

### Ethics statement

All procedures were in accordance with Animal Welfare Regulations and approved by the Institutional Animal Care and Use Committee at the Columbia University Medical Center. The study was reviewed and approved by the institutional review board at the Columbia University Medical Center.

### Reagents

ABT263 was purchased from Selleckchem (Houston, TX, U.S.A.). TIC10/ONC201 (angular form) was purchased from Sigma Aldrich (St. Louis, MO, U.S.A.). A 10 mM working solution in dimethylsulfoxide (DMSO) was prepared for both reagents prior to storage at −20°C.

### Cell cultures and growth conditions

T98G (*TP53* mut, *PTEN* mut) [50] human glioblastoma cells were obtained from the American Type Culture Collection (Manassas, VA, U.S.A.). NCH644 and NCH421K stem cell-like glioma cells were obtained from Cell Line Services (CLS, Heidelberg, Germany). The identities of the glioblastoma cell lines we purchased were confirmed by the respective source of purchase. SF188 (*TP53* mut, *PTEN* wt) [50] pediatric glioblastoma cells were kindly provided by Dr. Craig Thompson (Memorial Sloan Kettering Cancer Center, New York, NY, U.S.A.). MGPP-3 (PDGF(+), p53(−/−), PTEN(−/−)) is a murine proneural glioblastoma cell which was kindly provided by Dr. Peter Canoll. All cells were cultured as previously described [51]. Briefly, T98G and MGPP-3 cells were cultured in DMEM with 10% FBS, 4.5 g/L glucose, 4 mM L-glutamine, 1 mM pyruvate, 100 units/ml penicillin and 100 μg/ml streptomycin for maintenance. For experimental conditions these cells were cultured in DMEM containing only 1.5% FBS to mimick the nutrition starved environment within tumors. For the culture of SF188 the fore-mentioned medium was supplemented in addition with 2 mM L-alanyl-L-glutamine (GlutaMAX™-I, Gibco, Japan). NCH644 and NCH421K glioma stem-like cells were cultured in MG-43 medium (CLS, Heidelberg, Germany) for both maintenance and experiments.

### Cell viability assays

In order to examine cellular proliferation, 3-[4, 5-dimethylthiazol-2-yl]-2, 5-diphenyltetrazolium bromide (MTT) assays were performed as previously described [52, 53].

### Measurement of apoptosis and mitochondrial membrane potential

For annexin V/propidium iodide (PI) staining the FITC Annexin V Apoptosis Detection Kit I (BD Pharmingen, U.S.A.) was used according to the manufacturer's instructions. Staining for PI was performed as previously described [28]. The data were analysed with the FlowJo software (version 8.7.1; Tree Star, Ashland, OR, U.S.A.).

### Western blot analysis and co-immunoprecipitation

Specific protein expression in cell lines was determined by Western blot analysis as described before [28] using the following primary antibodies: Mcl-1 (1:500; CST: Cell Signaling Technology, Danvers, MA), human caspase-9 (1:1,000; CST), cleaved caspase-3 (1:250; CST), cleaved PARP (Asp214, 1:1000; CST), Bcl-xL (1:500; CST), Usp9X (1:1000; CST), pERK1/2 (1:500, CST), ERK1 (1:500, CST), pFoxo3a Ser253 (1:200, CST), Foxo3a (1:200, CST), BIM (1:500; CST), Noxa (1:500, clone 114C307; Calbiochem), β-actin (1:2,000, clone AC15; Sigma Aldrich), Bag3 (1:500; Abcam, Cambridge, MA). 14-3-3 (1:1,000, SCB: Santa Cruz Biotechnology), GAPDH (1:1000, clone 1D4, Novus Biologicals) and secondary HRP-linked antibodies were purchased from SCB.

For co-immunoprecipitations the Dynabeads® Co-Immunoprecipitation Kit (Novex, Life Technologies AG, Oslo, Norway) was used according to the manufacturer's instructions. Briefly, magnetic beads were conjugated either with monoclonal mouse anti Mcl-1 (clone 22) from SCB or with non-specific mouse IgG (SCB) overnight at 37°C. After washing the beads, lysates were added and incubated for 30 min at 4°C on a rotating wheel. Then, the beads were washed and the antibodies were eluted prior to removing the magnetic beads. Finally, the elution was resolved by SDS-PAGE and immunoblots were performed using the following primary antibodies: Mcl-1 (1:500; CST), Noxa (1:500, clone 114C307; Calbiochem) and BIM (1:500; CST).

### siRNA transfection

SignalSilence® Usp9X siRNA I #6308 was purchased from CST. Non-targeting siRNA-pool (ON-TARGETplus Non-targeting Pool, # D-001810-10-05) and siRNA against Bag3 (SMARTpool: ON-TARGETplus Bag3 siRNA, L-011957-00-0005), PMAIP1 siRNA and Mcl-1 (SMARTpool: ON-TARGETplus Mcl-1 siRNA, L-004501-00-0005) were purchased from Thermo Fisher Scientific (Pittsburgh, PA) and transfected as previously described [18, 54]. Briefly, cells were incubated for 6 h with the formed complexes of Lipofectamine® 2000 (Invitrogen, Carlsbad, CA, U.S.A.) and the respective siRNA (12-well condition) in DMEM without FBS and antibiotics. After 6 h, FBS was added to a total concentration of 1.5%.

### Real-time PCR and cDNA synthesis

RT-PCR was performed as described before [31] using the following primers: Usp9X forward: GTG TCA GTT CGT CTT GCT CAG C; Usp9X reverse: GCT GTA ACG ACC CAC ATC CTG A; Bag3 forward: TGC CAG AAA CCA CTC AGC CAG A; Bag3 reverse: TGA GGA TGA GCA GTC AGA GGC A; Mcl-1 forward: CCA AGA AAG CTG CAT CGA ACC AT; Mcl-1 reverse: CAG CAC ATT CCT GAT GCC ACC T; GAPDH forward: GTC TCC TCT GAC TTC AAC AGC G and GAPDH reverse: ACC ACC CTG TTG CTG TAG CCA A.

### Subcutaneous xenograft model

5 × 10^5^ MGPP-3 cells suspended 1:1 in Matrigel® (Corning Inc., Corning, NY, U.S.A.) were implanted subcutaneously into the flanks of 6–8 week-old SCID SHO mice as previously described [28]. Treatment was performed intraperitoneally 3 times a week for 2 weeks. For intraperitoneal application ABT263 and TIC10/ONC201 were dissolved in 80% Cremophor EL (SIGMA, St. Louis, MO) and 20% Ethanol (Pharmco-Aaper, Brookfield, CT) (v/v).

### Histological analysis

Subcutaneous tumors and samples from organs were extracted from SCID SHO mice and fixed for at least 24 h in 10% PBS-buffered formalin [28]. Then tissues were embedded in paraffin and 4 μm thick sections were cut prior to staining with hematoxylin and eosin. TUNEL staining was performed as previously described [18]. Microphotographs were taken at x40 magnification.

### Statistical analysis

Statistical significance was assessed by Student's *t*-test using Prism version 5.04 (GraphPad, La Jolla, CA, U.S.A.). A *p* ≤ 0.05 was considered statistically significant. The CompuSyn software (ComboSyn, Inc., Paramus, NJ, U.S.A. - http://www.combosyn.com last accessed 06/01/15) was used for the drug combination analysis including the calculation of the combination index (CI) and isobologram. A CI < 1 was considered as synergistic, a CI = 1 as additive and a CI > 1 as antagonistic. The concentration for each compound resulting in 50% inhibition (ED_50_) is normalized to 1, plotted on x- or y-axis and connected by a line which represents the ED_50_ isobologram. Data points of drug combinations plotted below the connecting line represent a synergistic interaction, data points located on the line represent an additive interaction and data points located above the connecting line represent an antagonistic interaction.

## SUPPLEMENTARY FIGURES


