# Supplementary material for: TIC10/ONC201 synergizes with Bcl-2/Bcl-xL inhibition in glioblastoma by suppression of Mcl-1 and its binding partners in vitro and in vivo
Source: Oncotarget. 2015 Oct 12;6(34):36456–71. doi: 10.18632/oncotarget.5505 (PMC4742189; doi:10.18632/oncotarget.5505)
Supplement: Supplementary file 1 [file oncotarget-06-36456-s001.pdf]

SUPPLEMENTARY FIGURES

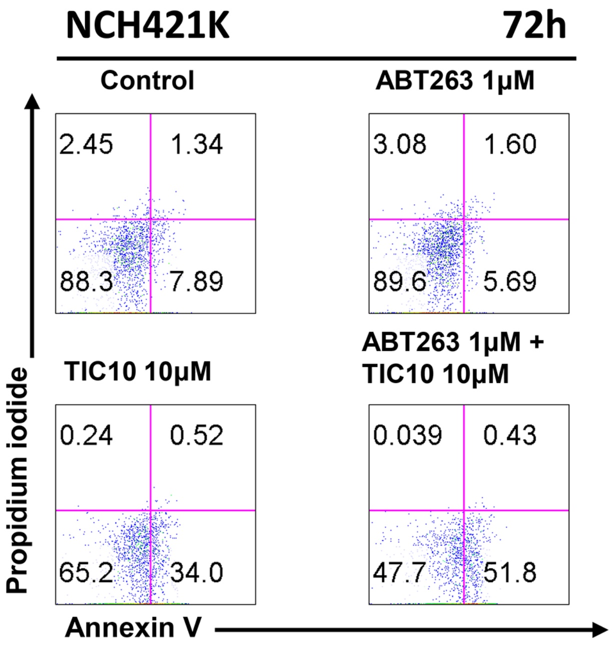

Supplementary Figure S1: Combined treatment with ABT263 and TIC10/ONC201 results in an enhanced induction of apoptosis in NCH421K glioma stem-like cells. Representative histograms of NCH421K glioma stem-like cells that were treated for 72 h with the indicated concentrations of TIC10/ONC201, ABT263, both or solvent prior to staining with propidium iodide and flow cytometric analysis.

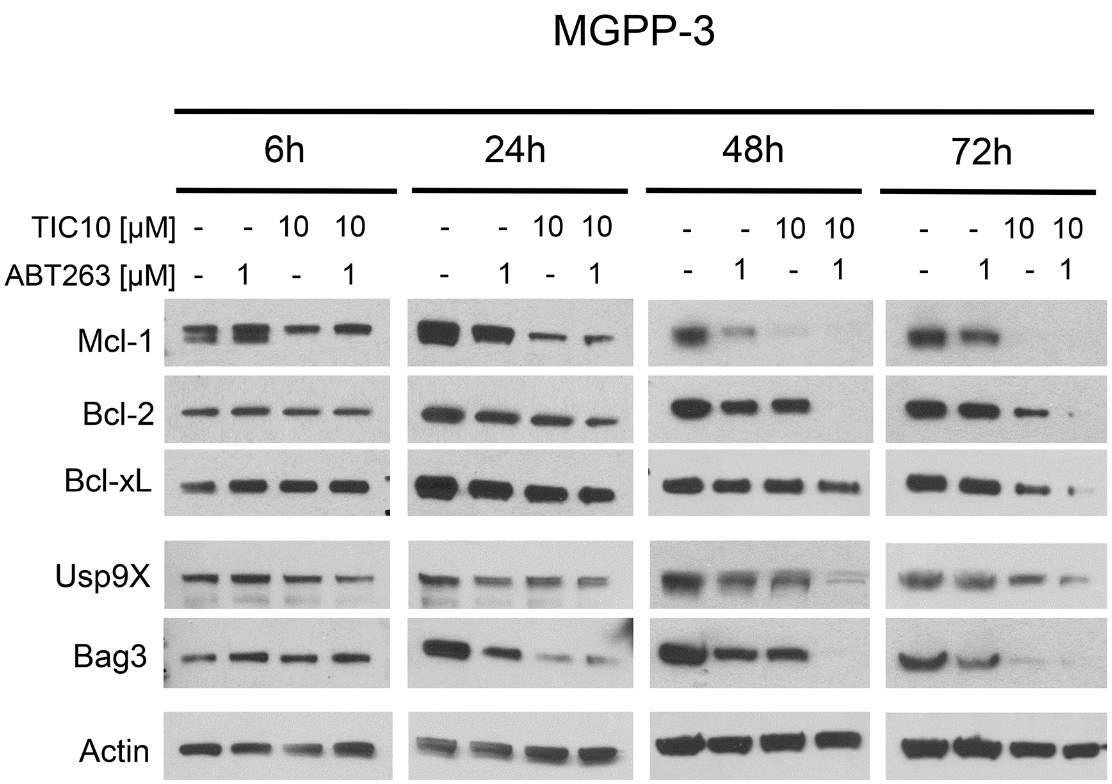

Supplementary Figure S2: Combined treatment with ABT263 and TIC10/ONC201 down-regulates the Mcl-1/Bag3/Usp9X network in proneural glioblastoma cells. MGPP-3 (PDGF(+), p53(-/-), PTEN(-/-)) glioblastoma cells that were derived from a murine transgenic model were treated for indicated lengths of time with ABT263, TIC10/ONC201, both agents or solvent under serum starvation (1.5% FBS). Whole-cell extracts were examined by Western blot for Mcl-1, Bcl-2, Bcl-xL, Bag3 and Usp9X. Actin served as a loading control.

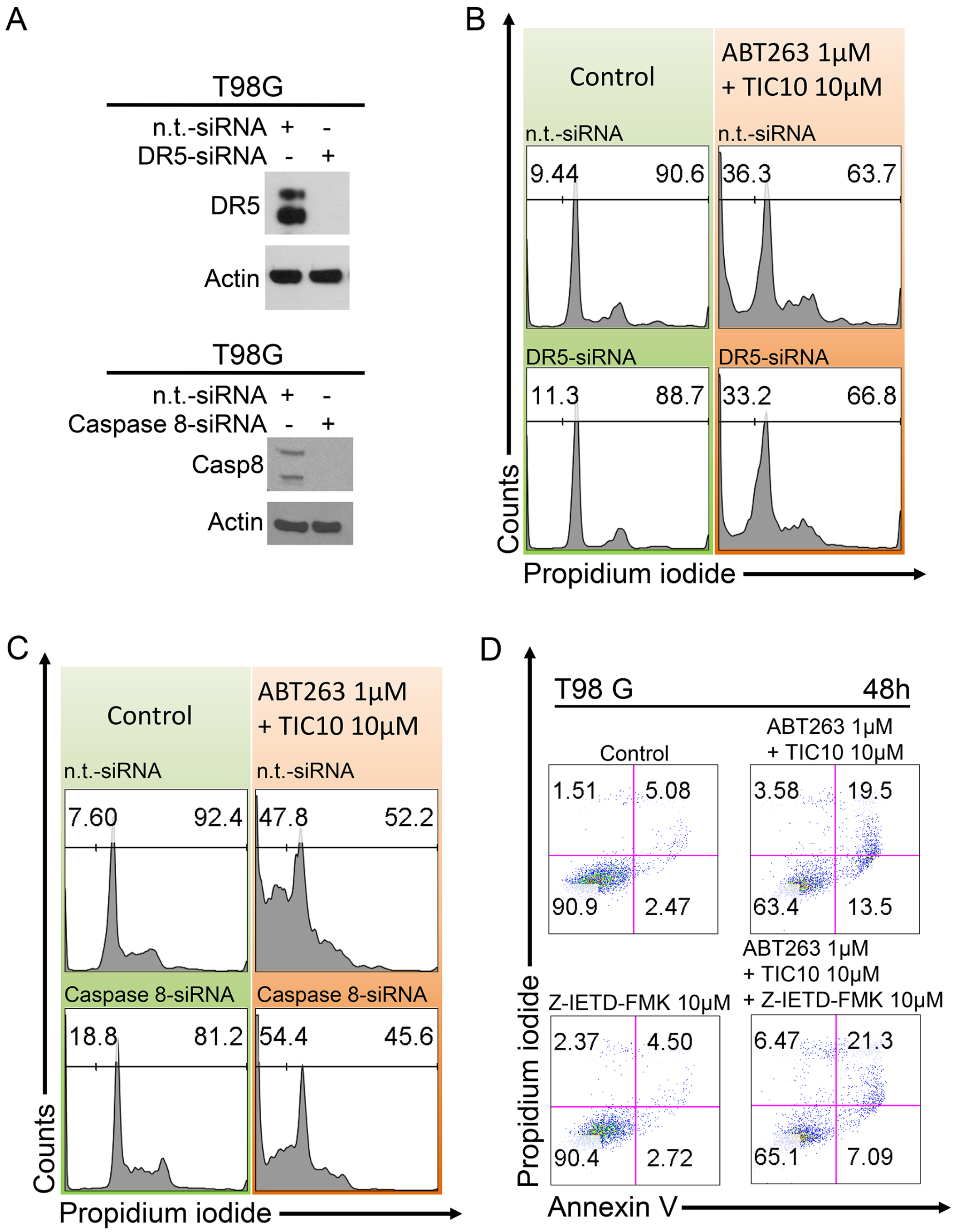

Supplementary Figure S3: The synergistic pro-apoptotic effect of ABT263 and TIC10/ONC201 is not mediated through the extrinsic pathway. A. T98G glioblastoma cells were transfected with non-targeting (n.t.)-siRNA, DR5-siRNA or caspase 8-siRNA. Whole-cell extracts were examined by Western blot for DR5 or caspase 8 (Casp8). Actin Western blot analysis was performed to confirm equal protein loading. **B.** T98G glioblastoma cells were transfected with non-targeting (n.t.)-siRNA or DR5-siRNA prior to treatment for 24 h with solvent or the combination of ABT263 and TIC10/ONC201 at indicated concentrations. Staining with propidium iodide and flow cytometric analysis was performed to determine the fraction of sub-G1 cells. **C.** T98G glioblastoma cells were transfected with non-targeting (n.t.)-siRNA or caspase 8-siRNA prior to treatment for 24 h with solvent or the combination of ABT263 and TIC10/ONC201 at indicated concentrations. Staining with propidium iodide and flow cytometric analysis was performed to determine the fraction of sub-G1 cells. **D.** T98G glioblastoma cells were treated for 48 h with solvent, Z-IETD-FMK or the combination of ABT263/TIC10 in the absence or presence of Z-IETD-FMK. Staining for annexin V/propidium iodide and flow cytometric analysis was performed to determine the fraction of apoptotic cells. Representative histograms are shown.

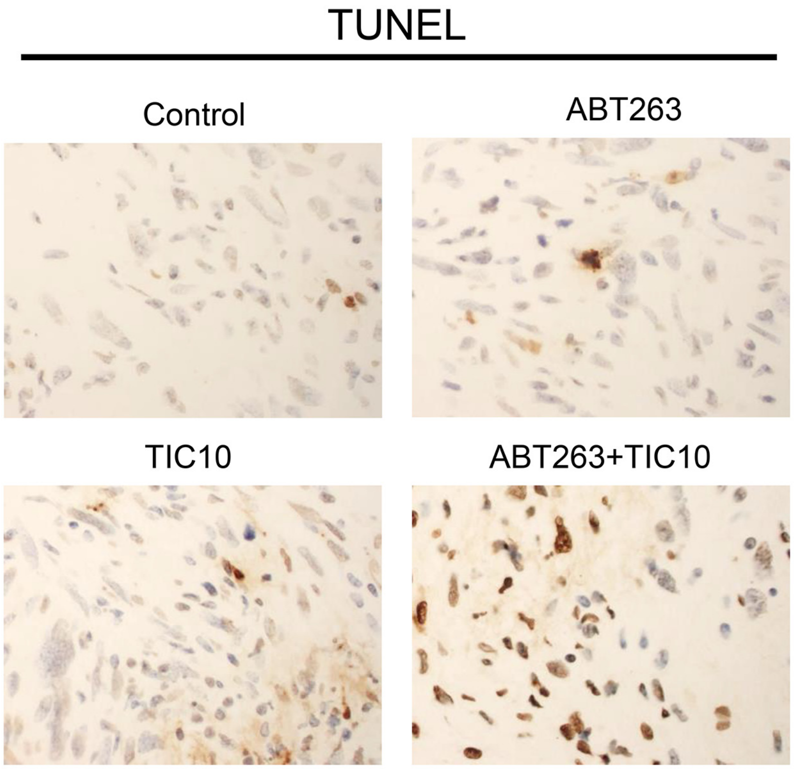

Supplementary Figure S4: Combined treatment with ABT263 and TIC10/ONC201 results in an enhanced induction of DNA-fragmentation. Tumors of animals treated with ABT263, TIC10/ONC201, both or vehicle were extracted and subjected to histological analysis. TUNEL staining was performed to detect DNA fragmentation.
